# Supplementary material for: Drinking-water 4,4′-methylenedianiline establishes a longitudinally tractable mouse model of cholestatic liver injury
Source: Toxicol Sci. 2026 Nov 2;209(11):kfag142. doi: 10.1093/toxsci/kfag142 (PMC13755879; doi:10.1093/toxsci/kfag142)
Supplement: Supplementary file 1 [file kfag142_supplementary_data.docx]

**Supplementary Information for “Drinking-Water 4,4′-Methylenedianiline Establishes a Longitudinally Tractable Mouse Model of Cholestatic Liver Injury”**

Takumi Iwasaka^1^, Katsuhisa Morita^1^, Iori Azuma^1^, Tomoka Nakagawa^1^, Eri Nakashima^1^, Tomomi Kamei^2^, Yuki Kato^2^, Hiroyuki Kusuhara^1^, Tadahaya Mizuno^3, †^

^1^Graduate School of Pharmaceutical Sciences, the University of Tokyo, Bunkyo-ku, Tokyo, 113-0033, Japan

^2^Laboratory for Drug Discovery and Development, SHIONOGI & Co.,Ltd. Toyonaka, Osaka, 561-0825, Japan,

^3^Graduate School of Pharmaceutical Sciences, the University of Tokyo, Bunkyo-ku, Tokyo, 113-0033, Japan, Electronic address: tadahaya@gmail.com

†Corresponding author: Tadahaya Mizuno

**Supplementary Methods**

**RNA-seq analysis**

Poly(A)-tailed mRNA was purified from total RNA prepared with ISOGEN II (311-07361, Nippon Gene, Japan) using the NEBNext Poly(A) mRNA Magnetic Isolation Module. RNA-seq libraries were prepared using the NEBNext Ultra II Directional RNA Library Prep Kit for Illumina. Libraries were sequenced using the NovaSeq 6000 S1 Reagent Kit v1.5 (200 cycles).

Quality control of reads was performed using PRINSEQ++ version 1.2.4 with the following parameters: trim_left=5, trim_tail_right=5, trim_qual_right=30, ns_max_n=20, and min_len=30. Transcript abundance was quantified using Salmon version 1.6.0 with GENCODE vM28 transcript annotations and the GRCm39 primary assembly genome (1). Salmon was run with validateMappings, gcBias, and seqBias options using a decoy-aware index (2). Transcript-level quantification files, quant.sf, were imported into R version 4.2.3 using tximport in Bioconductor. Scaled Transcript per million values were used for downstream analysis.

For the supplementary analysis, expression profiles of selected genes related to bile acid synthesis, hepatobiliary transport, and biliary epithelial cells were extracted from the existing liver RNA-seq dataset and displayed by treatment and sampling time. This analysis was descriptive.

**Histopathology**

The left lateral lobe of the liver was excised and collected for histological analysis into a 5 mL tube containing 4 mL of 10% neutral-buffered formalin. Samples were fixed for 24 hours, washed with PBS for 2 hours, and subsequently stored in 70% ethanol. Tissue processing, sectioning, and staining with hematoxylin and eosin (H&E) or Sirius Red were outsourced to Genostaff Inc.

Tissue sections were prepared by longitudinally slicing the left lateral lobe. Fixed liver tissues were subjected to a standard dehydration and paraffin embedding protocol. Samples were first immersed in 70% ethanol at 4 °C for 3 days, followed by sequential incubations in 80%, 90%, and 100% ethanol for 1 hour each at room temperature (RT).

Subsequent processing was performed using an automated tissue processor (CT-Pro20, Genostaff Inc.). Tissues underwent three consecutive immersions in 100% ethanol (1.5 hours each at RT), followed by incubation in a 1:1 mixture of 100% ethanol and G-Nox (xylene substitute, Genostaff Inc.) for 1.5 hours at RT. Samples were then immersed sequentially in three changes of G-Nox (1.5 hours each at RT), and finally in three changes of paraffin wax at 65 °C for 1.5 hours each.

Paraffin blocks were sectioned at a thickness of 4 µm and used for both H&E and Sirius Red staining.

For H&E staining, tissue sections were deparaffinized with xylene, rehydrated through a graded ethanol series, and rinsed in running tap water for 5 minutes, followed by distilled water. Sections were stained with hematoxylin (Sigma) for 3 minutes, rinsed in running tap water for 20 minutes, stained with eosin alcohol (Sigma) for 3 minutes, and then mounted with Malinol (#2040, Muto).

For Sirius Red staining, deparaffinized tissue sections were stained in Iron Hematoxylin Working Solution (Weigert's Iron Hematoxylin #4034, Muto Pure Chemicals) for 3 minutes at RT, treated with hydrochloric acid ethanol solution for 3 minutes, and rinsed in running tap water followed by distilled water. After treatment with 5% phosphomolybdic acid solution (#27615, Nacalai Tesque) for 2 minutes at RT, the sections were stained in saturated picric acid containing 0.1% Sirius Red (#3306, Muto) for 90 minutes at RT. They were then rinsed in 0.5% hydrochloric acid solution for 1 minute, dehydrated in three changes of 100% ethanol and xylene, and finally coverslipped with Malinol (#2040, Muto).

Stained sections were examined microscopically in a non-blind manner by two pathologists. One was a veterinary pathologist with 3 years of experience, and the other was a certificated veterinary/toxicology pathologist with over 15 years of experience. The histopathological scores were established as follows: 0 - no change; 1 - minimal change; 2 - mild; 3 - moderate change; 4 - marked change; 5 - severe change. Sirius Red was also used to semi-quantify the extent of fibrosis.

**Flow cytometry analysis**

The liver sample was dissociated using gentleMACS according to the manufacturer’s instructions. Unless otherwise noted, PBS containing 2% fetal bovine serum was used as “Wash Buffer” thereafter. The washed samples were centrifuged (50×g, 4 °C for 3 min) to eliminate hepatocytes and were subjected to ACK Lysis Buffer. ACK buffer was prepared by adding 8,024 mg of NH_4_Cl (A2037, TCI, Japan), 10 mg of KHCO3 (166-03275, FUJIFILM Wako, Japan), and 3.772 mg of EDTA 2Na · 2H2O (6381-92-6, Dojindo Laboratories, Japan) into 1 L pure water. The samples were washed with the Wash Buffer three times and then the samples were subjected to flow cytometry analysis. Flow cytometric analysis was performed with FACSCelesta (BD Biosciences, USA), and data were analyzed with FlowJo software (Treestar). Proportions of NK cells (CD45+/CD3−/NK1.1+), B cells (CD45+/B220+), CD4+ T cells (CD45+/CD3+/B220−/CD4+), CD8+ T cells (CD45+/CD3+/B220−/CD4−/CD8a+), NKT cells (CD45+/CD3+/B220−/CD4−/CD8a−/NK1.1+), γδT cells (CD45+/CD3+/B220−/CD4−/CD8a−/gdTCR+), eosinophils (CD45+/CD11b+/Siglec-F+), neutrophils (CD45+/CD11b+/Siglec-F−/Ly6G+/Tim4−), monocytes (CD45+/CD11b+/Siglec-F−/Ly6G−/Tim4−/Ly6C+), monocyte-derived macrophages (CD45+/CD11b+/Siglec-F−/Ly6G−/Tim4−/Ly6C−), and Kupffer cells (CD45+/CD11b+/Siglec-F−/Ly6G−/Tim4+/Ly6C−) were acquired via flow cytometry. The antibodies used are listed in Supplementary Table S7.

**Histopathological analysis with a deep learning model**

The deep learning framework PyTorch version 2.0.1 was employed for this study. Deep learning and feature extraction tasks were carried out utilizing the FUJITSU Supercomputer PRIMEHPC FX1000 and FUJITSU Server PRIMERGY GX2570 (Wisteria/BDEC-01) at the Information Technology Center, The University of Tokyo. Python (version 3.11.3) with open-source packages was utilized for all analyses except for deep learning.

Pathological images of the liver were collected from the Open TG-GATEs (TGGATEs) database, along with corresponding pathological finding type annotations and individual rat information from TogoDB. These images were acquired at 20× magnification using Aperio ScanScope AT. The dataset preparation involved excluding compounds not sampled at all specified time points and limiting samples to the highest compound concentration. Subsequently, one control sample per condition and one image per individual were randomly selected, resulting in a dataset of 6508 whole slide images (WSI) for the liver. The construction of the finding-based model followed our previous work on expression learning based on pathological findings (3), with some differences such as varying image sizes and multi-label learning. The Open TG-GATEs dataset was split into Train1 and Test subsets in a 4:1 ratio, further split into Train2 and Validation subsets in a 4:1 ratio. Weak labels were generated through multi-label training using patches of pathological images and corresponding labels from the Train2 subset, focusing on eight pathological findings: proliferation bile duct, ground glass appearance, increased mitosis, inclusion body intracytoplasmic, deposit pigment, single cell necrosis, vacuolization cytoplasmic, and swelling. The model predicted the probability of these findings in images of the validation subset, repeated five times to apply resulting labels to Train1 subset patches. Patches were extracted based on predicted probabilities, and a new model was trained using these patches and corresponding probabilities for training of the finding-based model.

The formalin-fixed liver specimens underwent standard processing procedures, including slicing into 4 μm sections, staining with H&E, and visualization using ZEISS Axio Scan.Z1. Subsequently, the WSI were partitioned into patches of 256×256 pixels, and feature extraction was performed on each patch using the aforementioned model. After extracting features from the fourth layer, one class support vector machine (sklearn, version 1.4.1) was employed to classify whether each patch was anomalous or not. Additionally, dimensionality reduction of the features was achieved through PCA and visualized. Details are available on our GitHub repository (https://github.com/mizuno-group/MDAdrinkingwater).

**Supplementary Figures and Tables**

**Fig. S1. Cumulative estimates of water consumption in Cohort 2.**

Cumulative bottle weight loss from Day 0 to each sampling endpoint was normalized by the number of animals in the corresponding cage or cages. Separate cages were used for different endpoints. These estimates were not corrected for evaporation or leakage. Average daily intake over the initial intervals ending on Days 1–4 is summarized in Table 2.

**Fig. S2. Plasma ALT changes during MDA-DW and TAA-DW administration in Cohort 1.**

Time-course changes in plasma alanine aminotransferase (ALT) levels are shown. Each line represents an individual animal followed through Day 28 (n = 4 controls, n = 6 TAA-DW, and n = 6 MDA-DW).

**Fig. S3. Plasma biochemical profiles in Cohort 2.**

Time-course changes in plasma biochemical values during the first 8 days of administration are shown. Bars represent mean ± SD, and dots represent individual animals. The x-axis is categorical and is not scaled according to elapsed time.

**Fig. S4. Additional hepatic immune-cell populations in Cohort 2.**

Violin plots show the proportions of immune-cell populations at each time point. Dots represent individual animals. The x-axis is categorical and is not scaled according to elapsed time. gdT, γδ T cells; MonMac, monocyte-derived macrophages; NKT, natural killer T cells; NK, natural killer cells. Statistical analysis used Kruskal–Wallis tests with Benjamini–Hochberg correction across all 55 population–time-point tests in Fig. 4B and Fig. S4, followed, where the adjusted P value was below 0.05, by Dunn’s pairwise tests with Holm correction within each population–time-point set. Asterisks indicate differences from the concurrent control group, which is included in the statistical analysis but not displayed. Hash marks indicate differences between MDA-DW and TAA-DW. One, two, and three symbols indicate Holm-adjusted P values < 0.05, < 0.01, and < 0.001, respectively.

**Fig. S5. Individual dimensionality-reduction maps used for META visualization of transcriptome data.**

Transcriptome profiles were embedded in two dimensions using each dimensionality-reduction method included in META visualization. Values shown in the panel titles indicate the specified hyperparameter values. PCA, principal component analysis; MDS, multidimensional scaling; t-SNE, t-distributed stochastic neighbor embedding; UMAP, uniform manifold approximation and projection; spc, spectral embedding; LLE, locally linear embedding.

**Fig. S6. Expression profiles of selected genes related to bile acid homeostasis and biliary epithelial cells.**

Liver RNA-seq expression profiles of *Slc10a1*, *Abcb11*, *Abcc3*, *Slc51b*, *Cyp7a1*, *Krt19*, *Abcc2*, and *Abcb4* are shown for Cohort 2 on Days 1, 2, 3, 4, and 8. Dots represent individual animals; lines and error bars show the mean ± SEM of log₂-transformed scaled TPM. Sample sizes were 3 to 4 by group and time point. Lines connect group means from separate groups of animals sampled at each time point. The x-axis is categorical and is not scaled according to elapsed time. Values are presented descriptively.

| **Supplementary Table S1: Sequences of qPCR primers** | | |
| --- | --- | --- |
| **Gene** | **Forward primer** | **Reverse primer** |
| *Acta2* | CCCAGACATCAGGGAGTAATGG | TCTATCGGATACTTCAGCGTCA |
| *Col1a1* | TAAGGGTCCCCAATGGTGAGA | GGGTCCCTCGACTCCTACAT |
| *Gapdh* | AGAACATCATCCCTGCATCC | CACATTGGGGGTAGGAACAC |
| *18S rRNA* | GTAACCCGTTGAACCCCATT | CCATCCAATCGGTAGTAGCG |

| **Supplementary Table S2: Blood biochemical values (Cohort 1)** | | | | | | | | | | |  |  |  |
| --- | --- | --- | --- | --- | --- | --- | --- | --- | --- | --- | --- | --- | --- |
|  |  |  | **day 7** | | | **day 14** | | | | **day 28** | | | |
| **No.** | **group** | **dissection** | **BW** | **ALT** | **AST** | **BW** | **ALT** | **AST** | **TBIL** | **BW** | **ALT** | **AST** | **TBIL** |
| 1 | Control | 14 | 21.25 | 27 | 52 | 22.40 | 24 | 29 | 0.1 |  |  |  |  |
| 2 | Control | 14 | 21.93 | 23 | 41 | 22.55 | 25 | 45 | 0.1 |  |  |  |  |
| 3 | Control | 14 | 20.15 | 28 | 42 | 20.83 | 24 | 27 | 0.1 |  |  |  |  |
| 4 | Control | 14 | 21.47 | 24 | 45 | 22.67 | 21 | 31 | 0.1 |  |  |  |  |
| 5 | Control | 28 | 20.43 | 25 | 40 | 21.50 | 33 | 36 |  | 23.78 | 24 | 32 | 0.1 |
| 6 | Control | 28 | 20.34 | 28 | 42 | 21.60 | 26 | 36 |  | 23.47 | 24 | 33 | 0.1 |
| 7 | Control | 28 | 20.30 | 24 | 44 | 22.17 | 24 | 40 |  | 23.92 | 25 | 31 | 0.1 |
| 8 | Control | 28 | 20.75 | 27 | 49 | 21.70 | 30 | 51 |  | 23.72 | 24 | 34 | 0.1 |
| 9 | TAA | 14 | 17.15 | 170 | 174 | 19.06 | 186 | 145 | 0.2 |  |  |  |  |
| 10 | TAA | 14 | 17.39 | 329 | 290 | 20.40 | 97 | 116 | 0.2 |  |  |  |  |
| 11 | TAA | 14 | 17.34 | 69 | 128 | 19.64 | 129 | 123 | 0.3 |  |  |  |  |
| 12 | TAA | 14 | 17.73 | 253 | 253 | 19.44 | 115 | 126 | 0.2 |  |  |  |  |
| 13 | TAA | 14 | 15.72 | 124 | 175 | 20.39 | 164 | 131 | 0.4 |  |  |  |  |
| 14 | TAA | 14 | 17.29 | 79 | 118 | 19.80 | 80 | 88 | 0.3 |  |  |  |  |
| 15 | TAA | 28 | 17.24 | 256 | 248 | 18.36 | 54 | 65 |  | 19.42 | 70 | 59 | 0.2 |
| 16 | TAA | 28 | 17.50 | 447 | 438 | 18.82 | 102 | 113 |  | 20.18 | 50 | 43 | 0.3 |
| 17 | TAA | 28 | 16.99 | 85 | 151 | 19.53 | 92 | 108 |  | 20.55 | 79 | 71 | 0.4 |
| 18 | TAA | 28 | 17.37 | 196 | 186 | 19.54 | 76 | 78 |  | 20.96 | 106 | 67 | 0.5 |
| 19 | TAA | 28 | 18.55 | 114 | 208 | 18.94 | 75 | 72 |  | 21.60 | 69 | 53 | 0.3 |
| 20 | TAA | 28 | 18.16 | 290 | 258 | 19.53 | 109 | 94 |  | 21.41 | 84 | 77 | 0.3 |
| 21 | MDA | 14 | 17.09 | 511 | 360 | 21.18 | 359 | 213 | 0.4 |  |  |  |  |
| 22 | MDA | 14 | 19.80 | 290 | 230 | 21.23 | 271 | 91 | 0.3 |  |  |  |  |
| 23 | MDA | 14 | 21.14 | 253 | 178 | 21.92 | 259 | 221 | 0.3 |  |  |  |  |
| 24 | MDA | 14 | 17.82 | 183 | 173 | 21.05 | 100 | 103 | 0.2 |  |  |  |  |
| 25 | MDA | 14 | 17.09 | 345 | 281 | 20.79 | 231 | 203 | 0.3 |  |  |  |  |
| 26 | MDA | 14 | 16.70 | 931 | 620 | 21.77 | 480 | 305 | 0.6 |  |  |  |  |
| 27 | MDA | 28 | 18.45 | 402 | 327 | 20.89 | 259 | 166 |  | 21.48 | 274 | 260 | 0.2 |
| 28 | MDA | 28 | 19.23 | 260 | 209 | 23.00 | 251 | 219 |  | 21.66 | 156 | 143 | 0.1 |
| 29 | MDA | 28 | 17.30 | 326 | 262 | 20.49 | 181 | 174 |  | 20.35 | 67 | 90 | 0.2 |
| 30 | MDA | 28 | 19.22 | 383 | 262 | 21.41 | 220 | 169 |  | 20.87 | 124 | 127 | 0.1 |
| 31 | MDA | 28 | 19.24 | 348 | 277 | 21.60 | 227 | 163 |  | 20.70 | 119 | 148 | 0.1 |
| 32 | MDA | 28 | 20.58 | 239 | 179 | 21.72 | 203 |  |  | 21.56 | 72 | 101 | 0.2 |

| **Supplementary Table S4a: Blood biochemical values (Cohort 2)** | | | | | | | | | | | |
| --- | --- | --- | --- | --- | --- | --- | --- | --- | --- | --- | --- |
| **No.** | **ID** | **group** | **day** | **BW** | **ALT** | **AST** | **ALP** | **TBIL** | **TCHO** | **HDL** | **TG** |
| 1 | 1C1 | Control | 1 | 22.11 | 21 | 30 | 195 | 0.1 | 63 | 56 | 130 |
| 2 | 1C2 | Control | 1 | 20.99 | 29 | 38 | 253 | 0.1 | 59 | 50 | 132 |
| 3 | 1C3 | Control | 1 | 22.18 | 20 | 31 | 160 | 0.2 | 62 | 56 | 109 |
| 4 | 1C4 | Control | 1 | 22.58 | 23 | 36 | 202 | 0.1 | 70 |  |  |
| 5 | 2C1 | Control | 2 | 21.22 | 25 | 32 | 182 | 0.2 | 62 | 57 | 219 |
| 6 | 2C2 | Control | 2 | 20.7 | 22 | 33 | 188 | 0.1 | 51 |  |  |
| 7 | 2C3 | Control | 2 | 21.42 | 22 | 33 | 249 |  |  |  |  |
| 8 | 3C1 | Control | 3 | 20.9 | 27 | 40 | 220 | 0.1 | 61 | 52 | 71 |
| 9 | 3C2 | Control | 3 | 20.69 | 28 | 37 | 184 | 0.2 | 61 | 52 | 85 |
| 10 | 3C3 | Control | 3 | 20.65 | 23 | 34 | 205 | 0.1 | 66 | 59 | 110 |
| 11 | 4C1 | Control | 4 | 21.24 | 23 | 35 | 176 | 0.1 | 53 | 48 | 81 |
| 12 | 4C2 | Control | 4 | 20.54 | 25 | 33 | 241 | 0.1 | 63 | 58 | 127 |
| 13 | 4C3 | Control | 4 | 20.03 | 23 | 32 | 195 | 0.3 | 57 | 51 | 114 |
| 14 | 4C4 | Control | 4 | 20.39 | 24 | 35 | 158 | 0.1 | 64 | 57 | 102 |
| 15 | 8C1 | Control | 8 | 19.98 | 25 | 37 | 163 | 0.1 | 64 | 52 | 64 |
| 16 | 8C2 | Control | 8 | 20.53 | 26 | 38 | 164 | 0.2 | 66 | 55 | 69 |
| 17 | 8C3 | Control | 8 | 21.41 | 26 | 40 | 171 | 0.2 | 62 | 63 | 63 |
| 18 | 8C4 | Control | 8 | 20.27 | 24 | 37 | 148 | 0.2 | 58 | 53 | 59 |
| 19 | 1T1 | TAA | 1 | 20.19 | 32 | 44 | 258 | 0.1 | 72 | 53 | 56 |
| 20 | 1T2 | TAA | 1 | 20.9 | 112 | 103 | 367 | 0.2 | 88 | 60 | 121 |
| 21 | 1T3 | TAA | 1 | 21.17 | 50 | 58 | 256 | 0.1 | 72 | 58 | 76 |
| 22 | 1T4 | TAA | 1 | 20.28 | 74 | 100 | 291 | 0.1 | 74 |  |  |
| 23 | 1T5 | TAA | 1 | 22.09 | 50 | 55 | 259 | 0.2 | 71 | 57 | 98 |
| 24 | 1T6 | TAA | 1 | 20.82 | 55 | 65 | 294 | 0.2 | 84 | 60 | 64 |
| 25 | 2T1 | TAA | 2 | 19.15 | 1600 | 1110 | 293 | 0.2 | 64 | 54 | 30 |
| 26 | 2T2 | TAA | 2 | 21.11 | 140 | 58 | 304 | 0.2 | 82 | 67 | 101 |
| 27 | 2T3 | TAA | 2 | 20.47 | 190 | 70 | 257 | 0.2 | 86 | 67 | 91 |
| 28 | 2T4 | TAA | 2 | 17.13 | 41800 | 37650 | 360 | 0.7 | 53 | 38 | 34 |
| 29 | 2T5 | TAA | 2 | 19.44 | 44950 | 25000 | 214 | 0.6 | 43 | 31 | 48 |
| 30 | 2T6 | TAA | 2 | 19.46 | 18550 | 21700 | 305 | 0.8 | 69 | 55 | 28 |
| 31 | 2T7 | TAA | 2 | 17.34 | 15650 | 9000 | 347 | 0.4 | 77 |  |  |
| 32 | 2T8 | TAA | 2 | 16.55 | 46700 | 40100 | 387 | 0.8 | 34 |  |  |
| 33 | 3T1 | TAA | 3 | 18.28 | 3880 | 1840 | 261 | 0.3 | 98 | 57 | 28 |
| 34 | 3T2 | TAA | 3 | 19.87 | 3680 | 2080 | 240 | 0.4 | 63 | 34 | 20 |
| 35 | 3T3 | TAA | 3 | 17.91 | 6520 | 5040 | 262 | 0.8 | 53 | 27 | 15 |
| 36 | 3T4 | TAA | 3 | 19.2 | 4020 | 1580 | 273 | 0.4 | 88 | 50 | 37 |
| 37 | 3T5 | TAA | 3 | 19.68 | 3680 | 1580 | 234 | 0.4 | 117 | 70 | 34 |
| 38 | 3T6 | TAA | 3 | 17.92 | 5000 | 3020 | 319 | 0.6 | 49 | 23 | 14 |
| 39 | 4T1 | TAA | 4 | 17.35 | 2476 | 2064 | 276 | 0.9 | 64 | 28 | 46 |
| 40 | 4T2 | TAA | 4 | 17.18 | 1510 | 1362 | 188 | 0.4 | 112 | 64 | 55 |
| 41 | 4T3 | TAA | 4 | 16.25 | 4920 | 5100 | 603 | 1 | 63 | 34 | 29 |
| 42 | 4T4 | TAA | 4 | 17.23 | 1232 | 1042 | 240 | 0.5 | 78 | 48 | 34 |
| 43 | 4T5 | TAA | 4 | 17.19 | 3930 | 4310 | 443 | 1 | 59 | 31 | 33 |
| 44 | 4T6 | TAA | 4 | 16.59 | 3108 | 2520 | 283 | 0.7 | 50 | 27 | 9 |
| 45 | 8T1 | TAA | 8 | 14.67 | 86 | 213 | 125 | 0.6 | 120 |  |  |
| 46 | 8T2 | TAA | 8 | 18.17 | 86 | 201 | 184 | 0.4 | 67 | 56 | 59 |
| 47 | 8T3 | TAA | 8 | 19.72 | 141 | 162 | 197 | 0.2 | 79 | 63 | 60 |
| 48 | 8T4 | TAA | 8 | 20.08 | 148 | 226 | 155 | 0.4 | 69 | 54 | 124 |
| 49 | 8T5 | TAA | 8 | 18.15 | 839 | 751 | 219 | 0.4 | 106 | 58 | 55 |
| 50 | 8T6 | TAA | 8 | 18.61 | 86 | 246 | 107 | 0.6 | 101 | 78 | 82 |
| 51 | 1M1 | MDA | 1 | 20.95 | 92 | 132 | 165 |  |  |  |  |
| 52 | 1M2 | MDA | 1 | 19.29 | 63 | 115 |  |  |  |  |  |
| 53 | 1M3 | MDA | 1 | 20.15 | 30 | 59 | 199 | 0.2 | 66 | 59 | 109 |
| 54 | 1M4 | MDA | 1 | 19.53 | 72 | 154 | 219 | 0.4 | 96 | 60 | 170 |
| 55 | 1M5 | MDA | 1 | 20.87 | 97 | 202 | 173 | 0.9 | 131 | 66 | 152 |
| 56 | 1M6 | MDA | 1 | 20.2 | 139 | 317 | 149 | 0.9 | 121 | 67 | 120 |
| 57 | 2M1 | MDA | 2 | 18.68 | 1500 | 2070 | 168 | 4.7 | 240 | 78 | 55 |
| 58 | 2M2 | MDA | 2 | 17.9 | 1985 | 2390 | 253 | 5.9 | 385 | 99 | 90 |
| 59 | 2M3 | MDA | 2 | 18.85 | 1015 | 1870 | 166 | 1 | 200 | 81 | 105 |
| 60 | 2M4 | MDA | 2 | 19.75 | 1145 | 1635 | 165 | 1.1 | 234 | 88 | 79 |
| 61 | 2M5 | MDA | 2 | 17.98 | 1415 | 1115 | 240 | 2.3 | 257 |  |  |
| 62 | 2M6 | MDA | 2 | 16.93 | 1360 | 2090 | 351 | 5.5 | 420 | 101 | 113 |
| 63 | 3M1 | MDA | 3 | 17.31 | 3370 | 2840 | 572 | 9 | 1080 | 120 | 67 |
| 64 | 3M2 | MDA | 3 | 18.19 | 610 | 470 | 277 | 2.1 | 405 |  |  |
| 65 | 3M3 | MDA | 3 | 17.41 | 900 | 730 | 294 | 5.5 | 640 | 150 | 76 |
| 66 | 3M4 | MDA | 3 | 15.76 | 3170 | 3100 | 634 | 12.1 | 1160 | 52 | 68 |
| 67 | 3M5 | MDA | 3 | 17.59 | 1000 | 940 | 509 | 8.6 | 1300 | 130 | 64 |
| 68 | 3M6 | MDA | 3 | 17.59 | 900 | 730 | 343 | 4.1 | 560 | 190 | 47 |
| 69 | 3M7 | MDA | 3 | 17.07 | 500 | 360 | 250 | 1.4 | 910 | 160 | 48 |
| 70 | 3M8 | MDA | 3 | 17.38 | 660 | 470 | 306 | 5.1 | 800 | 140 | 56 |
| 71 | 4M1 | MDA | 4 | 17.85 | 297 | 367 | 355 | 1.5 | 374 | 92 | 50 |
| 72 | 4M2 | MDA | 4 | 15.44 | 707 | 1070 | 867 | 3.9 | 1330 | 49 | 29 |
| 73 | 4M3 | MDA | 4 | 17.43 | 1108 | 1438 | 365 | 2.4 | 1160 | 120 | 57 |
| 74 | 4M4 | MDA | 4 | 16.31 | 648 | 791 | 522 | 4.3 | 2190 | 76 | 51 |
| 75 | 4M5 | MDA | 4 | 16.91 | 398 | 455 | 242 | 4.2 | 650 | 100 | 69 |
| 76 | 4M6 | MDA | 4 | 17.38 | 466 | 486 | 330 | 4.6 | 970 | 46 | 37 |
| 77 | 8M1 | MDA | 8 | 22.69 | 420 | 300 | 869 | 1.2 | 432 | 88 | 90 |
| 78 | 8M2 | MDA | 8 | 21.92 | 353 | 181 | 876 | 0.6 | 147 | 87 | 171 |
| 79 | 8M3 | MDA | 8 | 16.19 | 302 | 334 | 703 | 1.2 | 2040 | 200 | 40 |
| 80 | 8M4 | MDA | 8 | 20.04 | 565 | 313 | 1506 | 0.7 | 276 | 94 | 106 |
| 81 | 8M5 | MDA | 8 | 20.09 | 376 | 223 | 1458 | 1.3 | 1100 | 140 | 93 |
| 82 | 8M6 | MDA | 8 | 18.35 | 344 | 217 | 1158 | 0.8 | 268 | 87 | 42 |

| **Supplementary Table S3 (a): Histopathological Liver** Assessment Score (Cohort 1) |  |  |  |  |
| --- | --- | --- | --- | --- |
| **Control Group (sex:number)** |  | Male:4 | Male:4 | Male:4 |
| Administration Period (day) |  | 7 | 14 | 28 |
| Histopathology |  |  |  |  |
|  | Cellular infiltration, mixed inflammatory cell; focal |  |  |  |
|  | -Minimal | 0 | 0 | 2 |
| **TAA-DW group (sex:number)** |  | Male:4 | Male:3 | Male:4 |
| Administration Period (day) |  | 7 | 14 | 28 |
| Histopathology |  |  |  |  |
|  | Hypertrophy, hepatocyte; Centrilobular |  |  |  |
|  | -Minimal | 0 | 0 | 0 |
|  | -Mild | 3 | 1 | 2 |
|  | -Moderate | 1 | 2 | 2 |
|  | Necrosis, hepatocyte; Centrilobular |  |  |  |
|  | -Minimal | 2 | 1 | 4 |
|  | -Mild | 1 | 2 | 0 |
|  | Cellular infiltration, mononuclear inflammatory cell; Centrilobular |  |  |  |
|  | -Minimal | 0 | 3 | 4 |
|  | -Mild | 0 | 0 | 0 |
|  | -Moderate | 4 | 0 | 0 |
|  | Hyperplasia, oval cell; Centrilobular |  |  |  |
|  | -Minimal | 1 | 2 | 3 |
|  | -Mild | 0 | 0 | 1 |
|  | Mitosis, increased, hepatocyte |  |  |  |
|  | -Minimal | 0 | 0 | 0 |
|  | -Mild | 2 | 0 | 3 |
|  | Necrosis with hemorrhage, hepatocyte |  |  |  |
|  | -Minimal | 0 | 0 | 0 |
|  | -Mild | 1 | 0 | 0 |
|  | -Moderate | 2 | 0 | 0 |
|  | -Marked | 1 | 0 | 0 |
|  | Deposition, hemosiderin |  |  |  |
|  | -Minimal | 0 | 0 | 1 |
|  | -Mild | 0 | 1 | 2 |
|  | Fibrosis; Centrilobular |  |  |  |
|  | -Minimal | 0 | 1 | 1 |
| **MDA-DW group (sex:number)** |  | Male:4 | Male:4 | Male:4 |
| Administration Period (day) |  | 7 | 14 | 28 |
| Histopathology |  |  |  |  |
|  | Hyperplasia with degeneration, bile duct |  |  |  |
|  | -Minimal | 0 | 0 | 0 |
|  | -Mild | 3 | 0 | 1 |
|  | -Moderate | 1 | 4 | 3 |
|  | Fibrosis; Periportal |  |  |  |
|  | -Minimal | 0 | 1 | 3 |
|  | -Mild | 3 | 3 | 1 |
|  | -Moderate | 1 | 0 | 0 |
|  | Cellular infiltration, mixed inflammatory cell; Periportal |  |  |  |
|  | -Minimal | 0 | 0 | 3 |
|  | -Mild | 1 | 3 | 1 |
|  | -Moderate | 3 | 1 | 0 |
|  | Necrosis, hepatocyte; focal |  |  |  |
|  | -Minimal | 0 | 0 | 3 |
|  | -Mild | 3 | 1 | 0 |
|  | -Moderate | 1 | 3 | 1 |
|  | Aggregation, macrophage; focal |  |  |  |
|  | -Minimal | 0 | 1 | 4 |
|  | Mitosis, increased, hepatocyte |  |  |  |
|  | -Minimal | 3 | 1 | 0 |
|  | Atrophy, hepatocyte |  |  |  |
|  | -Minimal | 1 | 4 | 4 |
|  | -Mild | 3 | 0 | 0 |
| Numerals represent the number of animals with the findings |  |  |  |  |

| **Supplementary Table S3 (b): Histopathological scoring (control)** | | | |  |  |  |  |  |  |  |  |  |
| --- | --- | --- | --- | --- | --- | --- | --- | --- | --- | --- | --- | --- |
| Control |  |  |  |  |  |  |  |  |  |  |  |  |
| Animal Number | 1 | 2 | 3 | 4 | 5 | 6 | 7 | 8 | 9 | 10 | 11 | 12 |
|  | N | N | N | *N | *N | N | N | N | *N | A | N | A |
| Cellular infiltration, mixed cell, focal | ・ | ・ | ・ | ・ | ・ | ・ | ・ | ・ | ・ | 1 | ・ | 1 |

*: artifact

N=Normal; A=Abnormal; ・=Not Recorded

1：minimal

| **Supplementary Table S3 (c): Histopathological scoring (TAA)** | |  |  |  |  |  |  |  |  |  |  |  |
| --- | --- | --- | --- | --- | --- | --- | --- | --- | --- | --- | --- | --- |
| TAA |  |  |  |  |  |  |  |  |  |  |  |  |
| Animal Number | 1 | 2 | 3 | 4 | 5 | 6 | 7 | 8 | 9 | 10 | 11 | 12 |
|  | A | A | A | A | A | A | A | NE | A | A | A | A |
| Hypertrophy, hepatocyte, Centrilobular | 3 | 2 | 2 | 2 | 3 | 2 | 3 | ・ | 3 | 3 | 2 | 2 |
| Necrosis, hepatocyte, Centrilobular | 2 | 1 | ・ | 1 | 2 | 1 | 2 | ・ | 1 | 1 | 1 | 1 |
| Cellular infiltration, mononuclear cell, Centrilobular | 3 | 3 | 3 | 3 | 1 | 1 | 1 | ・ | 1 | 1 | 1 | 1 |
| Hyperplasia, oval cell, Centrilobular | ・ | 1 | ・ | ・ | ・ | 1 | 1 | ・ | 2 | 1 | 1 | 1 |
| Mitosis, increased, hepatocyte | ・ | 2 | ・ | 2 | ・ | ・ | ・ | ・ | ・ | 2 | 2 | 2 |
| Necrosis with hemorrhage, hepatocyte | 3 | 2 | 3 | 4 | ・ | ・ | ・ | ・ | ・ | ・ | ・ | ・ |
| Deposition, hemosiderin | ・ | ・ | ・ | ・ | ・ | 2 | ・ | ・ | 2 | 2 | 1 | ・ |
| Fibrosis, Centrilobular | ・ | ・ | ・ | ・ | ・ | 1 | ・ | ・ | 1 | ・ | ・ | ・ |

A=Abnormal; NE=Not Examine; ・=Not Recorded

1：minimal，2：mild，3：moderate，4：marked

| **Supplementary Table S3 (d): Histopathological scoring (MDA)** | | |  |  |  |  |  |  |  |  |  |  |
| --- | --- | --- | --- | --- | --- | --- | --- | --- | --- | --- | --- | --- |
| MDA |  |  |  |  |  |  |  |  |  |  |  |  |
| Animal Number | 1 | 2 | 3 | 4 | 5 | 6 | 7 | 8 | 9 | 10 | 11 | 12 |
|  | A | A | A | A | A | A | A | A | A | *A | A | A |
| Hyperplasia with degeneration, bile duct | 2 | 3 | 2 | 2 | 3 | 3 | 3 | 3 | 2 | 3 | 3 | 3 |
| Fibrosis, Periportal | 2 | 2 | 2 | 3 | 2 | 1 | 2 | 2 | 1 | 1 | 2 | 1 |
| Cellular infiltration, mixed cell, Periportal | 3 | 3 | 3 | 2 | 2 | 2 | 2 | 3 | 2 | 2 | 1 | 1 |
| Necrosis, hepatocyte, focal | 2 | 2 | 3 | 2 | 3 | 3 | 2 | 3 | 1 | 3 | 1 | 1 |
| Aggregation, macrophage, focal | ・ | ・ | ・ | ・ | ・ | ・ | 2 | ・ | 2 | 2 | 2 | 2 |
| Mitosis, increased, hepatocyte | 1 | 1 | ・ | 1 | ・ | 1 | ・ | ・ | ・ | ・ | ・ | ・ |
| Atrophy, hepatocyte | 2 | 2 | 2 | 1 | 1 | 1 | 1 | 1 | 1 | 1 | 1 | 1 |

*: artifact

A=Abnormal; ・=Not Recorded

1：minimal，2：mild，3：moderate

| **Supplementary Table S4b: Blood biochemical values (Cohort 2)** | | | | | | | | | |
| --- | --- | --- | --- | --- | --- | --- | --- | --- | --- |
| **day** | **Control** | | | **TAA** | | | **MDA** | | |
|  | **BW** | **ALT** | **AST** | **BW** | **ALT** | **AST** | **BW** | **ALT** | **AST** |
| 1 | 22.0±0.7 | 23.3±4.0 | 33.8±3.9 | 20.9±0.7 | 62.2±27.9 | 70.8±24.7 | 20.2±0.7 | 82.2±36.7 | 163.2±88.8 |
| 2 | 21.1±0.4 | 23.0±1.7 | 32.7±0.6 | 18.8±1.7 | 21197.5±20513.4 | 16836.0±16630.7 | 18.3±1.0 | 1403.3±336.8 | 1861.7±443.5 |
| 3 | 20.7±0.1 | 26.0±2.6 | 37.0±3.0 | 18.8±0.9 | 4463.3±1120.9 | 2523.3±1343.4 | 17.3±0.7 | 1388.8±1174.4 | 1205.0±1107.1 |
| 4 | 20.6±0.5 | 23.8±1.0 | 33.8±1.5 | 17.0±0.4 | 2862.7±1419.2 | 2733.0±1632.3 | 16.9±0.9 | 604.0±290.6 | 767.8±419.3 |
| 8 | 20.5±0.6 | 25.3±1.0 | 38.0±1.4 | 18.2±1.9 | 231.0±299.2 | 299.8±222.8 | 19.9±2.4 | 393.3±92.6 | 261.3±62.2 |

| **Supplementary Table S5: Result of rankproduct (MDA group)** | | | | |
| --- | --- | --- | --- | --- |
| **GO** | **log rank product statistic** | **p value** | **adjusted p value** |  |
| GOBP_RESPONSE_TO_INTERLEUKIN_17 | 23.59441201 | 1.72E-08 | 8.93357E-05 |  |
| GOBP_ACUTE_PHASE_RESPONSE | 21.39718743 | 1.28E-07 | 0.000333502 |  |
| GOBP_POSITIVE_REGULATION_OF_LEUKOCYTE_ADHESION_TO_VASCULAR_ENDOTHELIAL_CELL | 20.50336956 | 2.89E-07 | 0.000501057 |  |
| GOBP_POSITIVE_REGULATION_OF_MONOCYTE_CHEMOTAXIS | 19.00944453 | 1.11E-06 | 0.001143545 |  |
| GOBP_TOLL_LIKE_RECEPTOR_2_SIGNALING_PATHWAY | 18.8916615 | 1.24E-06 | 0.001143545 |  |
| GOBP_INTERLEUKIN_1_MEDIATED_SIGNALING_PATHWAY | 18.82266862 | 1.32E-06 | 0.001143545 |  |
| GOBP_PROTEIN_N_LINKED_GLYCOSYLATION_VIA_ASPARAGINE | 18.58377672 | 1.63E-06 | 0.001183214 |  |
| GOBP_NEGATIVE_REGULATION_OF_EXTRINSIC_APOPTOTIC_SIGNALING_PATHWAY_VIA_DEATH_DOMAIN_RECEPTORS | 18.4645133 | 1.82E-06 | 0.001183214 |  |
| GOBP_DENDRITIC_CELL_DIFFERENTIATION | 17.92885075 | 2.94E-06 | 0.001699703 |  |
| GOBP_T_HELPER_1_CELL_DIFFERENTIATION | 17.26202088 | 5.33E-06 | 0.002522101 |  |
| GOBP_FIBRINOLYSIS | 16.72306141 | 8.60E-06 | 0.003733369 |  |
| GOCC_COPI_COATED_VESICLE_MEMBRANE | 16.58094628 | 9.76E-06 | 0.003909202 |  |
| GOMF_MISFOLDED_PROTEIN_BINDING | 16.47659842 | 1.07E-05 | 0.003981687 |  |
| GOBP_POSITIVE_REGULATION_OF_PHAGOCYTOSIS_ENGULFMENT | 16.32898229 | 1.22E-05 | 0.004235156 |  |
| GOBP_REGULATION_OF_HETEROTYPIC_CELL_CELL_ADHESION | 15.42819574 | 2.70E-05 | 0.008270168 |  |
| GOBP_LEUKOCYTE_MIGRATION_INVOLVED_IN_INFLAMMATORY_RESPONSE | 15.28572009 | 3.06E-05 | 0.008684274 |  |
| GOBP_I_KAPPAB_PHOSPHORYLATION | 15.24587418 | 3.17E-05 | 0.008684274 |  |
| GOBP_ENDOPLASMIC_RETICULUM_TO_CYTOSOL_TRANSPORT | 14.7995492 | 4.68E-05 | 0.011614524 |  |
| GOBP_POSITIVE_REGULATION_OF_MACROPHAGE_CYTOKINE_PRODUCTION | 14.71111843 | 5.06E-05 | 0.011976983 |  |
| GOBP_ANTIFUNGAL_INNATE_IMMUNE_RESPONSE | 14.63301388 | 5.42E-05 | 0.012264544 |  |

| **Supplementary Table S6: Result of GAGE** | | | | |
| --- | --- | --- | --- | --- |
| **GO** | **treatment** | **time (h)** | **mean_diff** | **p_val** |
| GOBP_RESPONSE_TO_INTERLEUKIN_17 | TAA | 24 | 0.550283215 | 1.23085E-07 |
|  |  | 48 | 0.868088354 | 1.71702E-18 |
|  |  | 72 | 0.493649648 | 2.2926E-10 |
|  |  | 96 | 0.366445628 | 4.46203E-06 |
|  |  | 192 | 0.627270248 | 1.57E-07 |
|  | MDA | 24 | 0.861654153 | 7.13E-09 |
|  |  | 48 | 0.964777151 | 5.68E-18 |
|  |  | 72 | 0.775076157 | 1.90E-18 |
|  |  | 96 | 0.565623962 | 3.19171E-11 |
|  |  | 192 | 0.657065653 | 8.34029E-11 |
| GOBP_FIBRINOLYSIS | TAA | 24 | -0.188676844 | 0.024375036 |
|  |  | 48 | -0.61318091 | 4.0677E-05 |
|  |  | 72 | -0.787064263 | 7.15755E-05 |
|  |  | 96 | -0.925128835 | 7.91129E-08 |
|  |  | 192 | -0.206003079 | 7.83472E-08 |
|  | MDA | 24 | 0.69739285 | 2.97E-06 |
|  |  | 48 | 0.58491623 | 2.81E-06 |
|  |  | 72 | 0.59682587 | 6.07E-09 |
|  |  | 96 | -0.003638139 | 0.030206244 |
|  |  | 192 | -0.365541793 | 0.002067302 |

| **Supplementary Table S7: Antibodies used for FACS** | | | |
| --- | --- | --- | --- |
| **marker** | **Detection** | **ID** | **host** |
| CD45 | PE | 553081 | Rat |
| CD3 | FITC | 561798 | Rat |
| CD4 | BV786 | 563331 | Rat |
| CD8a | APC-Cy™7 | 561967 | Rat |
| B220 | BV480 | 565631 | Rat |
| NK1.1 | APC | 550627 | Rat |
| γδTCR | BV421 | 562892 | Rat |
| CD45 | R718 | 567075 | Rat |
| CD11b | BV480 | 566149 | Rat |
| Ly6C | FITC | 561085 | Rat |
| Ly6G | APC-Cy7 | 560601 | Rat |
| Tim4 | BV421 | 744874 | Rat |
| Siglec-F | Alexa Fluor® 647 | 562680 | Rat |

**References**

1.	Frankish A, Diekhans M, Ferreira A-M, Johnson R, Jungreis I, Loveland J, et al. GENCODE reference annotation for the human and mouse genomes. Nucleic Acids Res. 2019;47:D766–D773.

2.	Patro R, Duggal G, Love MI, Irizarry RA, Kingsford C. Salmon provides fast and bias-aware quantification of transcript expression. Nat Methods. 2017;14:417–419.

3.	Maedera S, Mizuno T, Kusuhara H. Investigation of latent representation of toxicopathological images extracted by CNN model for understanding compound properties in vivo. Comput Biol Med. 2024;168:107748.
